# A Zebrafish Model for Studies on Esophageal Epithelial Biology

**DOI:** 10.1371/journal.pone.0143878

**Published:** 2015-12-02

**Authors:** Hao Chen, Andrea Beasley, Yuhui Hu, Xiaoxin Chen

**Affiliations:** 1 Cancer Research Program, JLC-BBRI, North Carolina Central University, Durham, North Carolina, 27707, United States of America; 2 Center for Esophageal Diseases and Swallowing, Division of Gastroenterology and Hepatology, Department of Medicine, University of North Carolina at Chapel Hill, Chapel Hill, North Carolina, 27599, United States of America; University Medical Center of Princeton/Rutgers Robert Wood Johnson Medical School, UNITED STATES

## Abstract

Mammalian esophagus exhibits a remarkable change in epithelial structure during the transition from embryo to adult. However, the molecular mechanisms of esophageal epithelial development are not well understood. Zebrafish (*Danio rerio*), a common model organism for vertebrate development and gene function, has not previously been characterized as a model system for esophageal epithelial development. In this study, we characterized a piece of non-keratinized stratified squamous epithelium similar to human esophageal epithelium in the upper digestive tract of developing zebrafish. Under the microscope, this piece was detectable at 5dpf and became stratified at 7dpf. Expression of esophageal epithelial marker genes (Krt5, P63, Sox2 and Pax9) was detected by immunohistochemistry and *in situ* hybridization. Knockdown of P63, a gene known to be critical for esophageal epithelium, disrupted the development of this epithelium. With this model system, we found that Pax9 knockdown resulted in loss or disorganization of the squamous epithelium, as well as down-regulation of the differentiation markers Krt4 and Krt5. In summary, we characterized a region of stratified squamous epithelium in the zebrafish upper digestive tract which can be used for functional studies of candidate genes involved in esophageal epithelial biology.

## Introduction

The epithelial cells lining the human esophagus transform from simple columnar into ciliated epithelium at an early phase. The ciliated epithelium is then gradually replaced by squamous epithelium until a non-keratinized stratified squamous epithelium forms [1]. Although morphological changes during human esophageal development have been well characterized for several decades, the molecular mechanisms underlying this process are not fully understood [2]. Some of the genes and pathways critical for esophageal development, such as P63, Sox2, Nanog, Shh signaling and BMP signaling, are known to be important in esophageal diseases [3–6]. For example, P63 regulates growth, invasion and metastasis of esophageal squamous cell carcinoma (ESCC) cells [7], and Sox2 functions as an amplified lineage-survival oncogene in ESCC [8]. Additional research on esophageal development is needed to further describe the molecular mechanisms of esophageal diseases.

Our previous microarray study globally profiled genes and signaling pathways involved in the development of mouse esophagus, revealing novel players potentially critical for epithelial differentiation and maturation, such as Pax9, Nrf2, PPARβ/δ, and the PI3K/Akt pathway [9]. Pax9 is reportedly down-regulated in Barrett’s Esophagus, and in progressive loss in dysplastic and cancerous epithelium of the human esophagus [10]. Nevertheless, exact functions of these candidate genes need to be further elucidated. Although mice remain the first choice for such studies, zebrafish (*Danio rerio*) has attracted much attention as a model system for functional studies. The many favorable characteristics of zebrafish include convenient and economical genetic manipulations, short generation time, optical transparency of embryos, prolific reproduction, external development, and easy maintenance of both the adult and the young [11].

In this study, we aim to establish a zebrafish model for studies on esophageal epithelial biology. Previously we have identified a short piece of non-keratinized stratified squamous epithelium in adult zebrafish [12]. Here, we further characterized this epithelium in developing zebrafish and tested the feasibility of this model for investigating the functional roles of genes such as Pax9 in esophageal epithelial biology.

## Materials and Methods

### Zebrafish and morpholino (MO) knockdown

Zebrafish were obtained from Zebrafish International Resource Center (Eugene, OR). The AB strain was housed in an automatic zebrafish housing system (Aquaneering; San Diego, CA) at 28.5°C. All animal experiments were approved by the Institutional Animal Care and Use Committees at North Carolina Central University (protocol number: XC07-15-2008).

Three antisense MO oligonucleotides each of P63 and Pax9 (Gene Tools; Philomath, OR) were designed against exon/intron splice sites. MOs were solubilized in water at a concentration of 0.1–1.0 mM before injection into one to two-cell stage embryos. Preliminary data showed P63 MO-2 and Pax9 MO-1 were the most effective, and they were used for the subsequent knockdown experiments. P63 MO-2 was expected to block expression of all seven transcripts of zebrafish p63 gene. This MO blocked the splicing of intron 2–3 in 5 transcripts (tp63-201, tp63-001, tp63-002, tp63-004 and tp63-005). It also blocked the splicing of intron 4–5 in tp63-202, and intron 5–6 in tp63-003. The corresponding DNA sequence can be found at the following link: http://useast.ensembl.org/Danio_rerio/Gene/Summary?db=core;g=ENSDARG00000044356;r=6:28805219-28923664. Two specific mispair morpholinos were designed according to the sequences of the P63 MO-2 and Pax9 MO-1. These two mispair MOs and a scrambled control MO were synthesized by Gene Tools and then used as controls of MO toxicity and off-target effects. Mispair MOs were designed with the same sequence as the antisense MO, except at the five mispair sites. The sequences of the MOs were as follows:

P63 MO-1: 5’ TATACTGAGGCTGGAGGAAAAACAA 3’

P63 MO-2: 5’ ACATTTGCTGTATGTCTTACCGTCC 3’

P63 MO-3: 5’ GAGCATTGGTCTCCAGGTACAACAT 3’

Pax9 MO-1: 5’ AACATGAATATAGATCTTACCCATT 3’

Pax9 MO-2: 5’ TGGCTCTAGTTATGCAGATATATAA 3’

Pax9 MO-3: 5’ AAACTGAGAGGATGCTTACTTTGCT 3’

Scrambled control MO: 5'-CCTCTTACCTCAGTTACAATTTATA 3'

P63 mispair MO: 5’ AGATTTCCTGTATCTCTTAGCGTGC 3’

Pax9 mispair MO: 5’ AAGATCAATATACATGTTAGCCATT 3’

### Tissue samples and histochemical staining

Zebrafish were sacrificed at 4, 5, 6, 7 and 90 days post fertilization (dpf) and fixed with 4% paraformaldehyde. For adult zebrafish, the upper digestive tract was dissected and fixed with 4% paraformaldehyde. Whole zebrafish or tissue samples were orientated in agarose gel and processed for paraffin embedding. The paraffin blocks underwent serial sectioning (5 μm). Hematoxylin and eosin (H&E) staining was carried out with standard protocols. Alcian blue staining was performed as described previously [13]. Briefly, zebrafish at 7dpf were fixed with 4% paraformaldehyde and bleached with 30% hydrogen peroxide for 2 hours, and then washed with PBST and transferred into an Alcian blue solution (1% concentrated hydrochloric acid, 70% ethanol, 0.1% Alcian blue) and incubated overnight at room temperature. Specimens were then rinsed with acidic ethanol (5% concentrated hydrochloric acid, 70% ethanol). After these procedures, larval zebrafish were rehydrated in acidic ethanol of decreasing concentrations and finally cleared in glycerol in PBST.

### Immunohistochemistry (IHC)

Paraffin-embedded tissue sections were deparaffinized, rehydrated, and pretreated by heating the slides for 5–10 min in 10mM citrate buffer. Staining was performed with the ABC kit (Vector Laboratories) according to the manufacturer’s instructions with a mouse anti-P63 (4A4) antibody diluted at 1: 1000 (Cat # SC-8431, Santa Cruz Biotechnology; Dallas, Texas) and a rabbit anti-Sox2 antibody diluted at 1: 500 (Cat # ab97959, Abcam; Cambridge, MA).

### *In situ* hybridization (ISH)

Digoxigenin-labeled riboprobes (RNA probes) were synthesized as previously described [14]. DNA templates were amplified from zebrafish total RNA by RT-PCR with T7 promoter sequence in the antisense primer. Antisense RNA probes were generated for detection of Krt5, Krt4 and Pax9. The primers for generating the probes were:

Krt5 forward: 5' CTGCGTGAACTCCAGTCACAGAT 3'

Krt5 reverse: 5' TAATACGACTCACTATAGGGAGAAGCAATGCCAAGAAGATACA 3'

Krt4 forward: 5' GATGGACTGGGAAATGAGAAGAT 3'

Krt4 reverse: 5’ TAATACGACTCACTATAGGATAGCGTTTACTGCTGACGGTGG 3'

Pax9 forward: 5’ AAATGAATCCGACGTACTG 3’

Pax9 reverse: 5’ TAATACGACTCACTATAGGGAATGGCATGTCCACAGACAC 3’

The procedure of ISH on paraffin sections was based on the protocol of a commercial kit (IsHyb in situ Hybridization kit; Biochain; Newark, CA). The sections were washed and covered with cover slips for observation under the microscope.

### RT-PCR

To confirm the effect on splicing induced by P63 and Pax9 MOs, 50 zebrafish embryos injected with P63 MO-2, Pax9 MO-1 and control MO were collected at 3, 5 and 7 dpf. A Total RNA I kit (E.Z.N.A, Omega Bio-tek; Norcross, GA) was used to purify RNA, and the superscript reaction was carried out. Primers specific for P63 or Pax9 splicing variants were designed (P63 forward: CCAGCAGTCCAGCACAGCCAAAT, P63 reverse: CTCTGGTTTCCAATGTGACA; Pax9 forward: GACTCGGAACAGGTCAGAATAGG, Pax9 reverse: GAGTTGGATACGAATACAGGTGGTT). PCR using Advantage RT-for-PCR Kit (Clontech; Mountain View, CA) was carried out as follows: 95°Cx5’, (95°Cx1’, 60°Cx20”, 72°Cx1’30”) X 30 cycles, 72°Cx5’. The control PCR product is 449bp for P63 gene and 562bp for Pax9 gene, and MO injection resulted in un-spliced products of 758 bp for P63 gene and 1,011bp for Pax9 gene.

### Statistical analysis

GraphPad Prism 6.0e was used for one-way ANOVA analysis of all morphological data, with statistical significance level set at 0.05.

## Results

### Identification of a non-keratinized stratified squamous epithelium similar to human esophageal epithelium in zebrafish upper digestive tract

**Fig 1:**
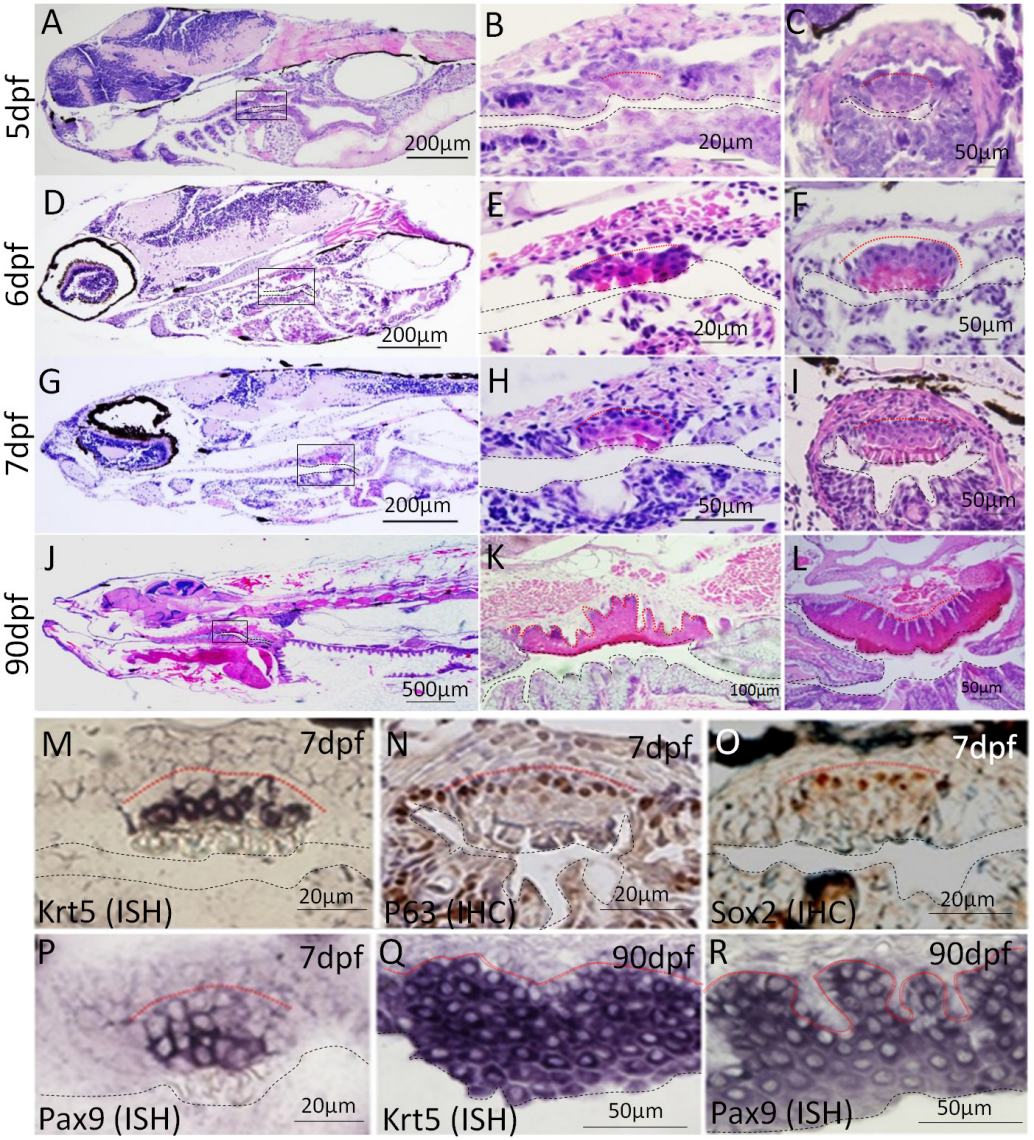
Identification of a non-keratinized stratified squamous epithelium in zebrafish upper digestive tract. (A-L) H&E staining of paraffin sections of zebrafish at 5, 6, 7 and 90 dpf shows the histogenesis of the squamous epithelium. A, D, G and J are the sagittal sections of the whole fish. B, E, H and K are magnifications of the areas in the yellow rectangles in A, D, G and J. Transverse sections show the histology of the squamous epithelium at 5, 6, 7 and 90 dpf (C, F, I, L). ISH for Krt5 (M, Q), IHC for P63 (N), IHC for Sox2 (O) and ISH for Pax9 (P, R) on transverse sections at 7dpf show the expression of esophageal genes in developing and adult zebrafish. All the pictures are dorsal side up. Base membrane of the squamous epithelium is marked with red dotted line and esophageal lumen is lined with black dotted line.

Twenty zebrafish were sacrificed each at 4, 5, 6 and 7dpf. H&E staining on paraffin sections of the whole zebrafish showed the digestive tract was thoroughly lined with simple columnar cells, except for a small piece of non-keratinized stratified squamous epithelium on the dorsal side of the upper digestive tract. This piece was detectable at 5dpf, stratified at 7dpf and histologically similar to human esophageal epithelium at 90dpf (Fig 1A–1L). At 5dpf, several round or larger square cells started to show up with distinct features on H&E-stained sections. These cells had pink cytoplasm and higher cytoplasm-to-nuclear ratios. The epithelium became more distinct at 6dpf by forming a compact cluster of pink cells on the surface. The cluster is organized into 4 cell layers at 7dpf; in sequential order, these are: basal layer, parabasal layer, superficial layer and dead cell layer. The basal layer is lined with small, round cells with very little cytoplasm. When differentiated, these cells became bigger and more square-shaped, with increased cytoplasm-to-nuclear ratios, to form the parabasal and superficial layers. A dead cell layer forms on the surface at 7dpf, which is believed to be shed off into the lumen. Similar to human esophageal epithelium, papillae were formed in adult zebrafish epithelium with multiple layers of parabasal and superficial cells. This epithelium is detectable by 5μm-serial sectioning in 18% of the fish at 5dpf, 80% at 6dpf and 100% at 7dpf (S1A Fig). The average number of total transverse sections containing this epithelium is 1.5 at 5dpf, 5.5 at 6dpf and 9.5 at 7dpf (S1B Fig).

The squamous epithelial genes Krt5 and P63 were expressed in the basal cells at 7dpf (Fig 1M and 1N). Two esophageal transcription factors, Sox2 and Pax9, are detectable at 7dpf (Fig 1O and 1P). We also detected abundant expression of Krt5 (Fig 1Q) and Pax9 (Fig 1R) in this epithelium in adult zebrafish. It is evident that, as of 7dpf, the histology and gene expression patterns of the non-keratinized stratified squamous epithelium in the zebrafish upper digestive tract are similar to those of human esophageal epithelium.

### P63 knockdown disrupted the development of the stratified squamous epithelium in zebrafish upper digestive tract

**Fig 2:**
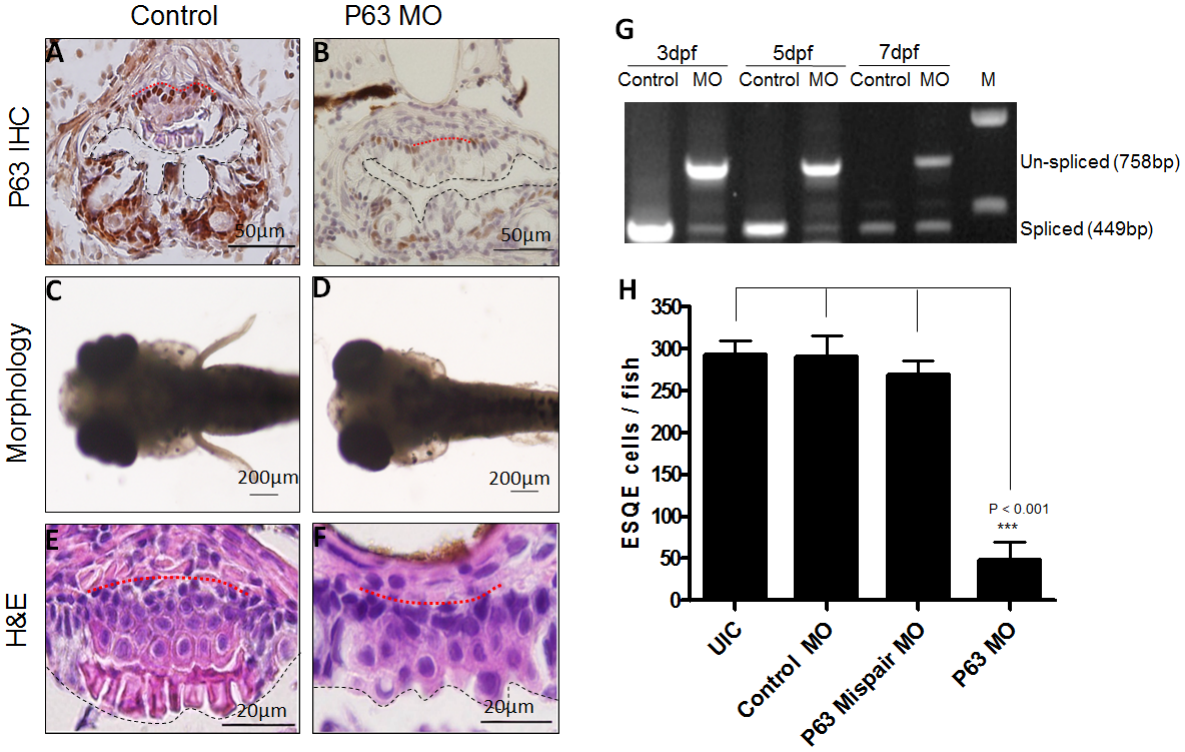
Phenotype of P63 knockdown zebrafish at 7dpf. (A) IHC for P63 protein on ESQE of zebrafish injected with control MOs. (B) IHC for P63 on ESQE of P63 knockdown zebrafish. (C) Normal zebrafish with pectoral fins (dorsal view). (D) Loss of pectoral fins in P63 knockdown zebrafish (dorsal view). (E) Normal ESQE. (F) Defective ESQE in a P63 knockdown zebrafish. (G) RT-PCR for P63 shows the un-spliced P63 transcript in MO injected zebrafish at 3, 5 and 7dpf. (H) Quantification shows a significant decrease of the number of ESQE cells in P63 knockdown zebrafish as compared with controls. All the pictures are dorsal side up. Base membrane of the squamous epithelium is marked with red dotted line and esophageal lumen is lined with black dotted line.

In order to examine the suitability of this squamous epithelium for molecular studies of esophageal epithelial development, we knocked down P63, a transcription factor known to be critical for mammalian esophageal development, using an antisense MO to interfere with mRNA splicing of P63 transcripts. A scrambled control and a P63 mispair control were used to control off-target effects. To validate the effect of MO on splicing, RT-PCR was performed with zebrafish at 3, 5 and 7dpf, to detect a 449bp spliced transcript and a 758bp un-spliced transcript. The spliced transcript was abundant at all stages in zebrafish injected with the control MO, but reduced in zebrafish injected with the antisense MO. On the contrary, in zebrafish injected with the antisense MO, the un-spliced transcript was abundant at 3dpf, and still detectable at 5dpf and 7dpf, suggesting that the MO knockdown effect lasted up to 7 days after injection (Fig 2G).

Consistent with the RT-PCR data, IHC showed that P63 protein was also reduced in the esophageal squamous epithelium (ESQE) at 7dpf as compared with that in control samples (Fig 2A and 2B). Furthermore, antisense MO caused loss of the pectoral fins (Fig 2C and 2D) in 67% of the injected zebrafish (S2B Fig), which was similar to the armless phenotype in P63 knockout mice[15]. Using serial sectioning and H&E staining, we found that P63 knockdown resulted in loss of the whole epithelium in 45% zebrafish (S3B Fig) and disappearance of the dead cell layer (Fig 2E and 2F) in 55% zebrafish (S2C Fig). In these zebrafish there were a total of 2–3 sections per zebrafish containing ESQE as compared with 8–10 sections in control zebrafish (S2D Fig). In addition, the number of squamous epithelial cells was also significantly reduced in P63 knockdown zebrafish as compared to controls (Fig 2H). These data indicate that the zebrafish ESQE responded to genetic manipulation and may be used for screening genes critical for esophageal development.

### Pax9 knockdown impaired the differentiation of the stratified squamous epithelium in zebrafish upper digestive tract

Our previous microarray studies suggested Pax9 as a critical transcription factor for esophageal development [9]. Previous studies also reported an association of Pax9 with esophageal diseases such as Barrett’s esophagus and ESCC [10, 16]. In the current study, we detected Pax9 expression in ESQE of developing and adult zebrafish (Fig 1F and 1H). We then knocked down Pax9 with a specific Pax9 antisense MO interfering with Pax9 mRNA splicing in order to investigate the role of Pax9 in esophageal development.

**Fig 3:**
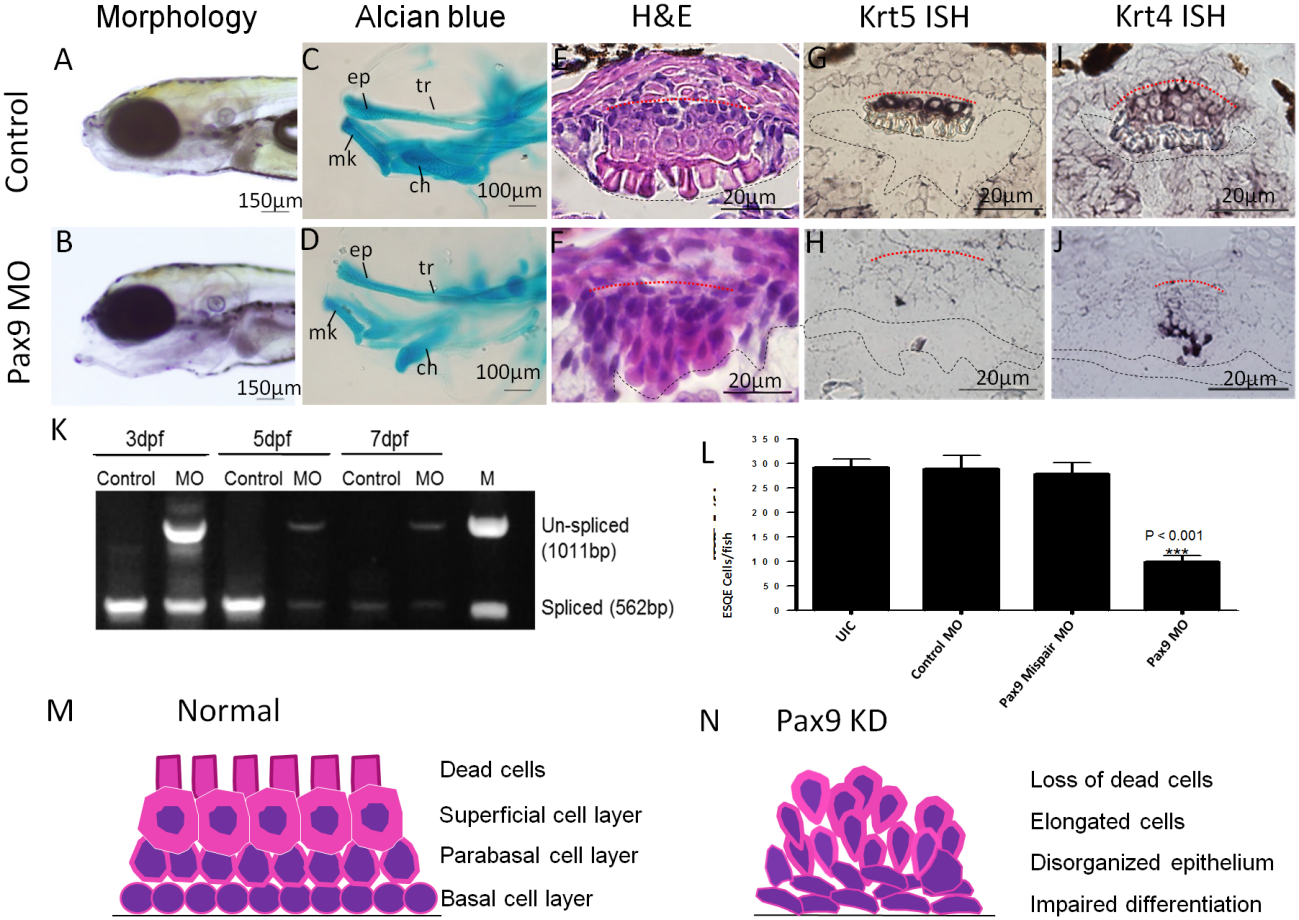
Expression of Pax9 in zebrafish ESQE and phenotypes of Pax9 knockdown zebrafish at 7dpf. (A) Normal zebrafish (Lateral view). (B) Pax9 knockdown fish with malformation of the lower jaw (Lateral view). (C) Alcian blue staining shows the orofacial cartilage of normal zebrafish (lateral view). (D) Alcian blue staining shows the malformed cartilage of Pax9 knockdown zebrafish (lateral view). (E) Normal ESQE in zebrafish. (F) Disorganized ESQE in a Pax9 knockdown zebrafish. (G) Krt5 ISH on normal zebrafish ESQE. (H) Krt5 ISH on Pax9 knockdown zebrafish ESQE. (I) Krt4 ISH on normal zebrafish ESQE. (J) Krt4 ISH on Pax9 knockdown zebrafish ESQE. (K) RT-PCR for Pax9 shows the un-spliced Pax9 transcript in MO injected zebrafish at 3, 5 and 7dpf. (L) Quantification shows a significant decrease of the number of ESQE cells in Pax9 knockdown zebrafish as compared with controls. (M) A schematic cartoon of normal zebrafish ESQE at 7dpf. (N) A schematic cartoon of Pax9 knockdown zebrafish ESQE at 7dpf. ep, ethmoid plate; ch, ceratohyal; mk, Meckel’s cartilage; tr, trabeculae. All the pictures are dorsal side up. Base membrane of the squamous epithelium is marked with red dotted line and esophageal lumen is lined with black dotted line.

A scrambled MO and a Pax9 mispair MO were used to control off-target effects. RT-PCR was performed with zebrafish at 3, 5 and 7dpf, to detect a 562bp spliced transcript and a 1,011bp un-spliced transcript. The spliced transcript was abundant at all the stages injected with control MO, but was reduced by the antisense MO. In the antisense MO injected zebrafish, the un-spliced transcript was abundant at 3dpf and still detectable at 7dpf, suggesting a lasting effect of antisense MO (Fig 3K). As expected, Pax9 knockdown resulted in defects of the jaw, hypomandibular cartilage and palatal skeleton (Fig 3A, 3B, 3C and 3D), which was similar to the phenotype in Pax9 knockout mice [17]. Moreover, Pax9 knockdown resulted in a loss of the epithelium in 52% of zebrafish (S2C Fig), and a disorganized epithelium (elongation of epithelial cells and loss of the dead cell layer) in 48% of zebrafish (Fig 3E and 3F) (S2C Fig). In those zebrafish with ESQE, the number of squamous epithelial cells also significantly decreased in Pax9 knockdown zebrafish as compared with controls (Fig 3L). This epithelium was found on 3–4 sections per zebrafish in Pax9 knockdown zebrafish as compared with 8–10 sections in control zebrafish (S2D Fig). In addition, Pax9 knockdown impaired the differentiation of ESQE as evidenced by down-regulation of differentiation markers, Krt5 and Krt4 (Fig 3G, 3H, 3I and 3J). A schematic cartoon is drawn to show the phenotype of ESQE in Pax9 knockdown zebrafish at 7dpf (Fig 3M and 3N).

## Discussion

Zebrafish have been a model organism for development and diseases in the intestine, but not in the esophagus, probably due to the fact that its entire digestive system, including the esophagus, is lined with simple columnar epithelium [18, 19]. Only one study on esophageal development has been performed in zebrafish, focusing on the embryonic stage (<74hpf), when the epithelium is still columnar [20]. If there is no stratified squamous epithelium in zebrafish esophagus, then it is not a suitable model organism for studying esophageal epithelial development.

Our study demonstrates there is actually a stratified squamous epithelium located on the dorsal side of zebrafish upper digestive tract between the pharynx and the intestinal bulb. Histologically, it is highly similar to the non-keratinized squamous epithelium in human esophagus, with basal, parabasal, and superficial layers. It is detectable under the microscope at 5dpf and becomes stratified at 7dpf. Expression of esophageal epithelial genes (Krt5, P63, Sox2 and Pax9) confirms its squamous features and endodermal origin. We speculate that this piece of squamous epithelium once fully developed may mainly function as a protective layer in adult zebrafish. P63 is exclusively detected in the basal layer of this epithelium in zebrafish esophagus, which is consistent with the fact that P63 is expressed in the basal cells of mammalian esophageal epithelium. In P63 knockout mice, the esophagus is lined with a simple columnar epithelium which fails to develop into stratified squamous epithelium [15]. Similarly, knocking down P63 in zebrafish resulted in a thinner and less squamous epithelium, suggesting the feasibility of this zebrafish model for developmental study. With this model, we performed a functional study on Pax9, which was identified as a potentially critical gene in esophageal development and diseases. Our data showed that Pax9 knockdown in zebrafish impaired differentiation of ESQE, supporting an important role of Pax9 in the pathogenesis of Barrett’s esophagus and ESCC. Given the fact that genomic knockout of Pax9 in mouse resulted in postnatal death with no visible phenotype [17], we generated a Krt5Cre;Pax9^fl/fl^ mouse line for conditional knockout of Pax9 in esophageal epithelium. This mouse line survives, and its esophageal phenotypes were similar to those in Pax9 knockdown zebrafish (data not shown).

This study opens up a new avenue for future studies on esophageal biology. The explosive growth of next-generation sequencing provides a rapidly increasing database of candidate genes involved in esophageal diseases [21, 22]. Mice and rats are the common model organisms for functional characterization of genes identified by genome sequencing projects. Although these models have significant advantages, they are expensive to maintain, relatively difficult to genetically manipulate, and not desirable for genetic screening. We believe the zebrafish model avoids these drawbacks and nicely complements mammalian experimental models.

In summary, we characterized a stratified squamous epithelium in the zebrafish upper digestive tract as a novel model system for studies on esophageal biology. With the application of MO technology and CRISPR/Cas9 technology [23], this model can be used to screen candidate genes *in vivo*, laying a foundation for further studies with mammalian model organisms.

## Supporting Information

S1 Fig Quantitation of the esophageal squamous epithelium in developing zebrafish. Percentage of the zebrafish (A) and the number of sections per zebrafish (B) with ESQE in the upper digestive tract at different developmental stages.
(JPG)

S2 Fig Survival curves and quantitation of P63 or Pax9 knockdown phenotypes. (A) Survival curves (up to 7dpf) after MO injection; (B) Pax9 morphants (malformed lower jaw) and P63 morphants (finless) at 7dpf; (C) Pax9 and P63 morphants with esophageal SQ epithelium at 7dpf; (D) Sections containing ESQE per fish at 7dpf after different MO injection. * P<0.05; ** P<0.01 as compared with UIC (un-injected control) or Control MO.
(JPG)

S3 Fig H&E staining on serial transverse sections containing all the esophageal squamous epithelium of 7dpf zebrafish. The sections show the histology of pharynx (A), esophagus (B-O) and intestine (P). There are 9 sections containing the stratified squamous epithelium (C-K). Scare bar:5μm.
(TIF)

## Abbreviations

dpf: days post fertilization
ESCC: esophageal squamous cell carcinoma
ESQE: esophageal squamous epithelium
H&E: hematoxylin and eosin
IHC: immunohistochemistry
ISH: *in situ* hybridization
MO: morpholino
