# Supplementary figures and images for: A Zebrafish Model for Studies on Esophageal Epithelial Biology
Source: PLoS One. 2015 Dec 2;10(12):e0143878. doi: 10.1371/journal.pone.0143878 (PMC4667901; doi:10.1371/journal.pone.0143878)

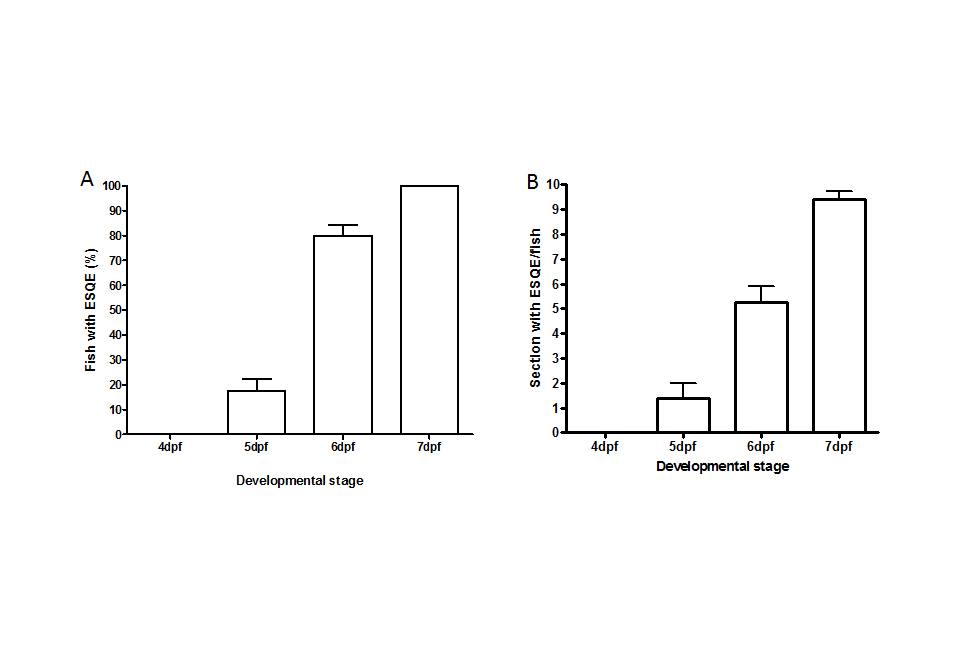

Supplement: S1 Fig — Percentage of the zebrafish (A) and the number of sections per zebrafish (B) with ESQE in the upper digestive tract at different developmental stages. (JPG) [file pone.0143878.s001.jpg]

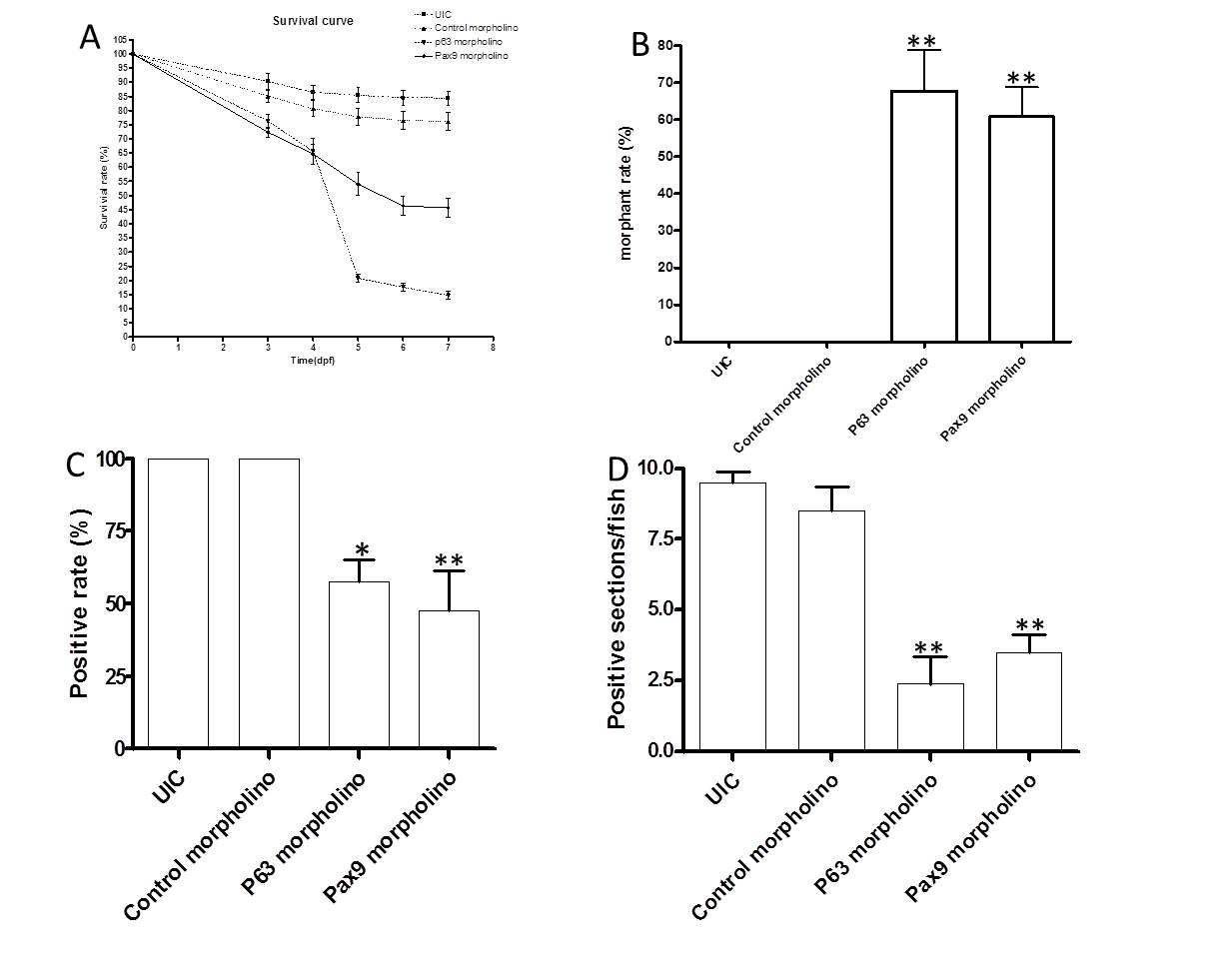

Supplement: S2 Fig — (A) Survival curves (up to 7dpf) after MO injection; (B) Pax9 morphants (malformed lower jaw) and P63 morphants (finless) at 7dpf; (C) Pax9 and P63 morphants with esophageal SQ epithelium at 7dpf; (D) Sections containing ESQE per fish at 7dpf after different MO injection. * P<0.05; ** P<0.01 as compared with UIC (un-injected control) or Control MO. (JPG) [file pone.0143878.s002.jpg]

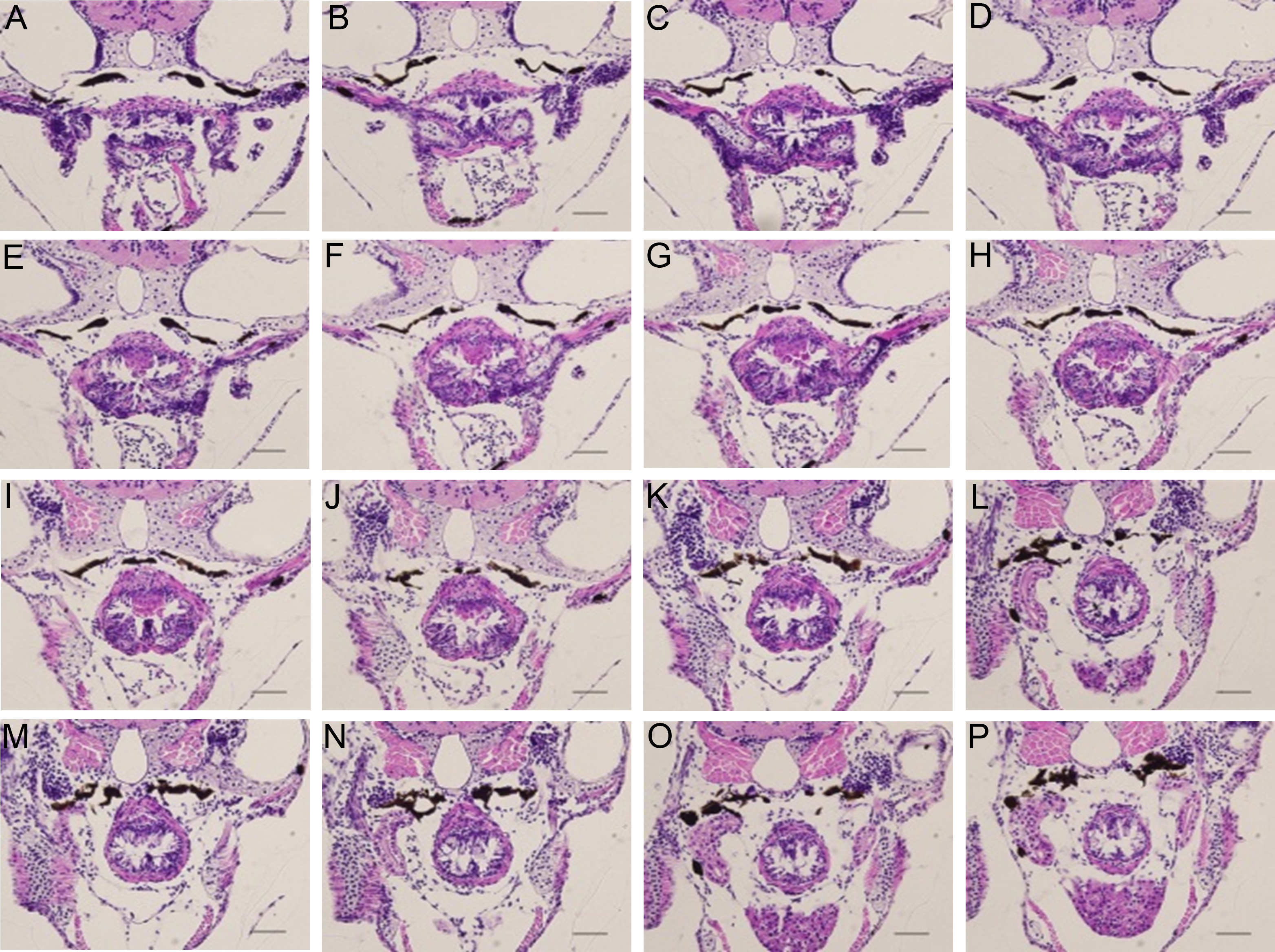

Supplement: S3 Fig — The sections show the histology of pharynx (A), esophagus (B-O) and intestine (P). There are 9 sections containing the stratified squamous epithelium (C-K). Scare bar:5μm. (TIF) [file pone.0143878.s003.tif]
